# Phylogeography of *Libanotis buchtormensis* (Umbelliferae) in Disjunct Populations along the Deserts in Northwest China

**DOI:** 10.1371/journal.pone.0159790

**Published:** 2016-07-21

**Authors:** Ping Wang, Xianzhi Zhang, Nan Tang, Jianjun Liu, Langran Xu, Kai Wang

**Affiliations:** 1 College of Forestry, Northwest A&F University, Yangling, Shaanxi, China; 2 Plateau Flower Research Centre, Qinghai University, Xining, Qinghai, China; 3 College of Landscape Architecture and Arts, Northwest A&F University, Yangling, Shaanxi, China; 4 College of Life Science, Northwest A&F University, Yangling, Shaanxi, China; National Cheng-Kung University, TAIWAN

## Abstract

In Northwest China, aridification and desert expansion play significant roles in promoting desert plant diversification and speciation. However, to date, little is known about the effects of the desert barrier on the population structure of montane, non-desert species in the area. In this study, we sequenced chloroplast DNA regions (*trn*L–*trn*F and *trn*S–*trn*G) and a nuclear gene (*rpb*2) to investigate the population differentiation and phylogeographical history of *Libanotis buchtormensis*, a perennial montane species possessing a disjunct distribution at the periphery of the central desert. In total, 23 chloroplast haplotypes and 24 nuclear haplotypes were recovered from the 21 natural populations and six hebarium specimens. Phylogenetic analysis based on the combined plastid and nuclear dataset revealed two distinct lineages of *L*. *buchtormensis*, which inhabit the disjunct areas on both sides of the desert zone. The molecular dating analysis indicated that the divergence between the southeastern and the northwestern populations occurred in the middle Pleistocene, concomitantly with the desert expansion. The geographical vicariance likely contributed to the present disjunct distribution of *L*. *buchtormensis* across the deserts in Northwest China. Populations in the southeastern region may have migrated from the northwestern region, and seem to be a peripheral distribution of *L*. *buchtormensis*.

## Introduction

**Fig 1:**
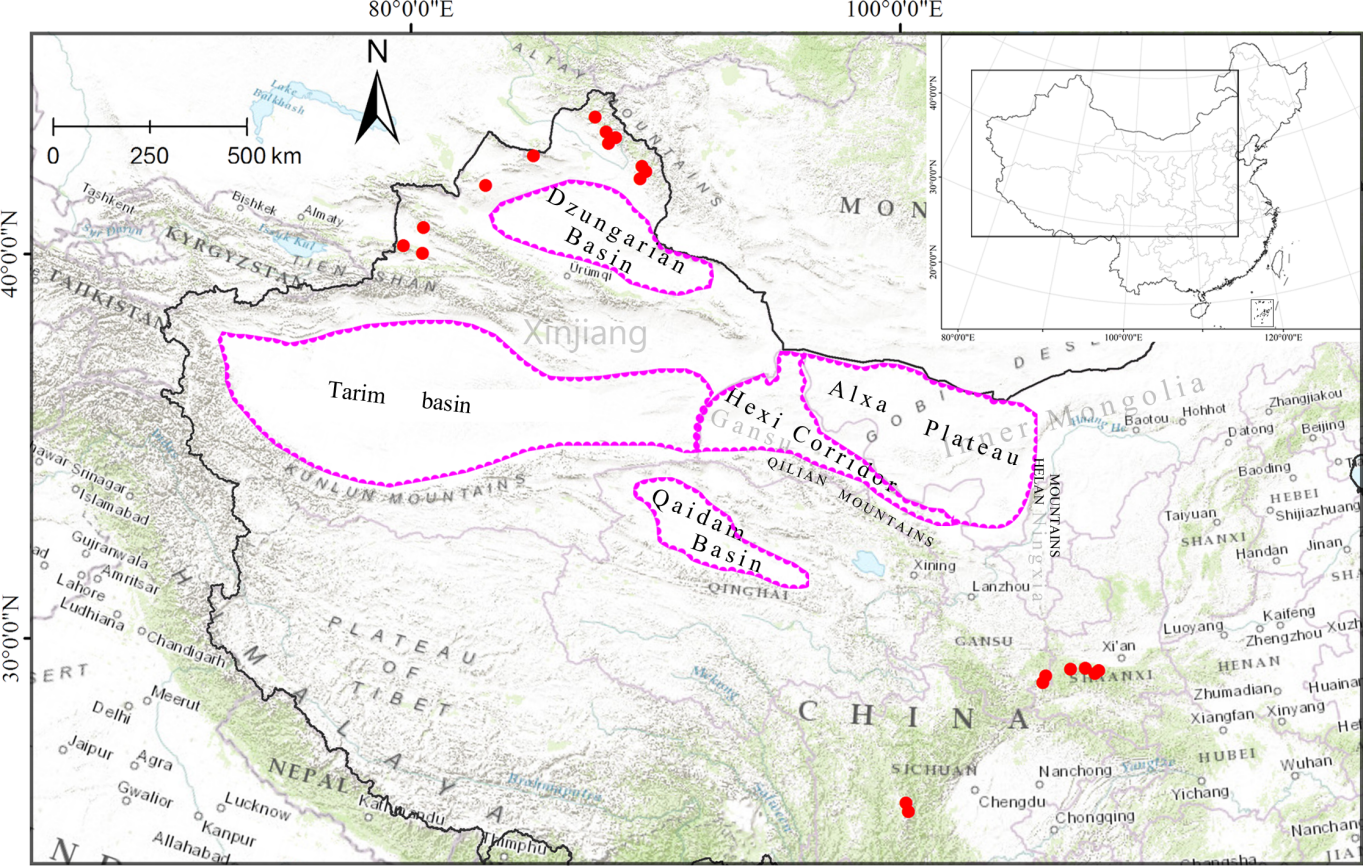
Locations of sampled populations (1–21) of *Libanotis buchtormensis* (with red dots, see Table 1 for population codes). The pink dashed line represents the main location of the desert zone in Northwest China according to Zhao (1985) [2].

Located in central Eurasia, the arid Northwest China (35°30'–49°N, 73–106°E) is one of the most arid zones in the world, covering the western parts of the Inner Mongolia Autonomous Region and Ningxia Provinces, across the Hexi Corridor in Gansu Province to the Xinjiang Uygur Autonomous Region (Fig 1) [1,2]. In contrast to other continuous arid zones in Africa, Western Asia, and Australia, the arid Northwest China is surrounded by high mountains: the Altai Mountains to the north–west, the Tienshan Mountains to the west, the Kunlun Mountains to the south–west, the Qilian Mountains to the south–east, and the Helan Mountains to the east. These mountains divide the arid zone into five major topographical sub–regions: the Dzungarian Basin, the Tarim Basin, the Qaidam Basin, the Hexi Corridor, and the Alxa Plateau, where sandy and stony deserts dominate (Fig 1) [2]. Far from the sea and surrounded by mountains, the climate of these sub–regions is hyper–arid (the annual precipitation of some areas is less than 60 mm). Under the influence of the continental dry air mass, only shrubs, subshrubs, and herbs that are able to tolerate extreme drought inhabit these regions [2,3]. However, the climate of the high–altitude mountain ranges (e.g. the Tienshan and Altai Mountains) is more humid due to occasional orographic precipitation [4,5].

Based on the varied geography and climate and on the plant diversity, the flora of the arid Northwest China is generally classified into the Asian desert flora subkingdom and the Eurasian forest subkingdom [3,6]. With global Pleistocene cooling, deserts expanded dramatically, which, together with aridification in the same period, profoundly affected the distributions and evolution of native plants [3,7]. The extremely dry deserts, such as the Bardain–Jaran Desert and Tengger Desert, act as barriers by obstructing the gene flow of plant species. Recent phylogenetic and phylogeographical studies show that desert development has promoted population differentiation and vicariant speciation of several desert species in the arid Northwest China [3,8–10]. However, to date, few phylogeographical studies have been performed for species distributed in the surrounding mountains, which belong to the Eurasian forest flora subkingdom.

*Libanotis buchtormensis* (Fisch.) DC. (Umbelliferae) presents an interesting model for studying the intraspecific divergence and spatiotemporal population dynamics of montane species in the arid Northwest China. This perennial herb is widely distributed in the mountain regions of Central Asia, and western and eastern Siberia [11,12]. *Libanotis buchtormensis* prefers sunny rocky slopes at 700–3000 m altitude [13]. *Libanotis buchtormensis* is insect–pollinated and mainly gravity–dispersed [14]. The species displays a peripheral desert distribution pattern in China, namely the northwestern region (NW), encompassing the Altai Mountains, Tienshan Mountains, and steppe mountains from the Altai to the Western Tienshan Mountains, and the southeastern region (SE), covering the Qinling Mountains and the western Sichuan Plateau. The geographic distance between the populations spanning the deserts is approximately 2800 km. Our previous study detected a strong geographical pattern of genetic differentiation among populations in the NW and SE regions based on ISSR data [14]. The desert zone likely acted as a geographical barrier to hamper the gene flow between the natural populations of *L*. *buchtormensis*. Moreover, morphological differences between the two regions have been observed [15]. Individuals in the NW region have coriaceous leaves and elliptical fruits, whereas their SE counterparts have papery leaves and obovate fruits [15].

In this study, we used maternally inherited chloroplast DNA sequences (*trn*L–*trn*F and *trn*S–*trn*G) and a bi–parentally inherited nuclear gene (*rpb*2) to investigate the phylogeographical history of *L*. *buchtormensis* distributed alongside the desert zone in Northwest China. The main goals were to answer the following questions: (1) How does the desert zone in Northwest China contribute to the genetic variation among *L*. *buchtormensis* populations? (2) When did the NW and SE populations diverge and expand/contract? and (3) What are the main driving forces for the disjunction of *L*. *buchtormensis*?

## Materials and Methods

### Ethics statement

No specific permits were required for the field studies in China described here because all researchers collecting samples had introduction letters from the College of Forestry, Northwest A&F University, Shaanxi. Neither endangered nor protected species were sampled during field sampling.

### Sampling

In total, 256 individuals from 21 populations were sampled, covering the possible geographical range of *L*. *buchtormensis* in China (Table 1, Fig 1). A decline in population size can be observed in its natural populations located in the Qinling Mountain and Sichuan region due to anthropogenic exploration. To better resolve the biogeography of *L*. *buchtormensis*, six available DNA samples extracted from herbarium specimens and representing the Qinling Mountains (three) and the Western Sichuan Plateau (three) were also used in the study. We used *Libanotis spodotrichom* K. F. Fu and *Peucedanum praeruptorum* Dunn as outgroups [13,16]. Voucher specimens of all populations sampled were deposited at the Herbarium of Northwest A&F University (WUK).

**Table 1:** Sample information and haplotypes recovered for 21 natural populations and 6 herbarium specimens of *L*. *buchtormensis*.

**Code**	Sample Location	*h*_cp_ (±SD)	*π*_cp_ (±SD)	Plastid haplotype frequencies	*h*_nr_ (±SD)	*π*_nr_ (±SD)	**Nuclear haplotype frequencies**	Voucher
**Overall**		0.909 (0.009)	0.0033 (0.00055)	22	0.886 (0.012)	0.00373 (0.0009)	24	
**NW**		0.921 (0.009)	0.00291 (0.00055)	18	0.883 (0.013)	0.00328 (0.0008)	18	
1. KT1	Koktokay, Xinjiang	0.485 (0.106)	0.00038 (0.00026)	C2(8), C3(4)	0.439 (0.158)	0.00164 (0.00076)	H3(2), H4(9), H5(1)	XJ2012001
2. KT2	Koktokay, Xinjiang	0	0	C2(5)	0	0	H1(5)	XJ2012002
3. KT3	Koktokay, Xinjiang	0	0	C1(10)	0.533 (0.095)	0.00123 (0.00058)	H1(6), H2(4)	XJ2012003
4. AT1	Altay, Xinjiang	0.467 (0.132)	0.00108 (0.00047)	C4(7), C5(3)	0	0	H6(10)	XJ2012004
5. AT2	Altay, Xinjiang	0	0	C6(10)	0	0	H7(10)	XJ2012005
6. AT3	Altay, Xinjiang	0.356 (0.123)	0.0011 (0.00042)	C5(2), C7(8)	0	0	H6(10)	XJ2012006
7. AT4	Altay, Xinjiang	0.448 (0.134)	0.00069 (0.00048)	C8(11), C9(3), C10(1)	0.533 (0.126)	0.00123 (0.00061)	H6(10), H8(3), H9(2)	XJ2014001
8. BJ	Burqin, Xinjiang	0.699 (0.049)	0.00171 (0.00051)	C5(5), C6(5), C11(7)	0.75 (0.068)	0.00173 (0.00059)	H9(7), H10(6), H11(2), H12(2)	XJ2012007
9. HB	Hoboksar, Xinjiang	0	0	C12(15)	0	0	H13(15)	XJ2012008
10. TL	Toli, Xinjiang	0.356 (0.081)	0.00055 (0.00039)	C13(2), C14(8)	0	0	H1(10)	XJ2012009
11. YN	Yining, Xinjiang	0.556 (0.095)	0.00215 (0.00061)	C15(5), C16(5)	0.721 (0.152)	0.00176 (0.001)	H1(2), H14(1), H15(6), H16(1)	XJ2012010
12. YL	Yili, Xinjiang	0.6 (0.109)	0.00146 (0.00061)	C5(9), C16(3), C17(3)	0.514 (0.069)	0.00178 (0.00061)	H1(6), H14(9)	XJ2014002
13. TK	Tekes, Xinjiang	0.485 (0.106)	0.0015 (0.00051)	C5(8), C18(4)	0.621 (0.087)	0.00141 (0.00066)	H15(5), H17(6), H18(1)	XJ2012011
**SE**		0.606 (0.033)	0.00085 (0.00026)	4	0.561 (0.048)	0.0017 (0.00058)	6	
14. ZD1	Kangding, Sichuan	0	0	C19(15)	0.343 (0.128)	0.00079 (0.0005)	H19(12), H20(3)	SC2012012
15. ZD2	Kangding, Sichuan	0	0	C19(15)	0.514 (0.069)	0.00118 (0.0005)	H19(9), H21(6)	SC2012013
16. HH	Heihe, Shaanxi	0.2	0.00015	C21(1), C22(9)	0.356 (0.159)	0.00041 (0.00041)	H22(8), H23(2)	SX2012014
17. HZ	Houzhenzi, Shaanxi	0	0	C21(15)	0	0	H22(15)	SX2012015
18. TBX	Taibai, Shaanxi	0.356 (0.159)	0.00028 (0.00027)	C22(8), C23(2)	0	0	H22(10)	SX2012016
19. TB	Taibai, Shaanxi	0	0	C22(15)	0.476 (0.092)	0.00055 (0.00035)	H22(10), H24(5)	SX2012017
20. HX	Huixian, Gansu	0	0	C22(15)	0.248 (0.131)	0.00028 (0.00035)	H22(13), H24(2)	GS2012018
21. LD	Liangdang, Gansu	0	0	C22(10)	0	0	H22(10)	GS2012019
**Herb.**								
Herb. 1	Kangding, Sichuan			C20				WUK34215
Herb. 2	Kangding, Sichuan			C20				WUK247646
Herb. 3	Kangding, Sichuan			C20				WUK300790
Herb. 4	Taibai, Shaanxi			C5				WUK131744
Herb. 5	Huixian, Gansu			C5				WUK102334
Herb. 6	Tianshui, Gansu			C5				WUK364419

*h*_cp_, haplotype diversity of cpDNA, *π*_cp_, nucleotide diversity of cpDNA, *h*_nr_, haplotype diversity of nuclear DNA, *π*_nr_, nucleotide diversity of nuclear DNA.

### DNA extraction, PCR amplification and sequencing

Genomic DNA was extracted from silica–gel dried leaf material based on the modified CTAB method [17]. Two plastid intergenic spacers, *trn*L–*trn*F and *trn*S–*trn*G, were amplified and sequenced in all *L*. *buchtormensis* samples (including herbarium specimens) following Taberlet et al. (1991) [18] and Hamilton (1999) [19]. PCR amplification was performed in a 25–μL reaction mixture following the program: 3 min initial denaturation at 94°C, then 35 cycles of 1 min denaturation at 94°C, 1 min annealing at 52°C, and 1 min extension at 72°C, ending with a 10 min final extension at 72°C. The *rpb*2, i.e., the 23rd intron of RNA polymerase beta subunit II, was amplified to provide nuclear DNA (nrDNA) polymorphisms at the population and possibly individual level. This region has been used successfully in phylogenetic and phylogeographic studies of various plant taxa [20–22]. Initial sequences were amplified using previously published universal 7F and 10R primers [21]. To sequence all the samples, two sequence–specific primer combinations were designed from the initial sequences. The primers used were forward ‘LB1F’: TTG TGT ATA AGT CAT GCC AAC and reverse ‘LB1R’: TTA AGT TTA GAA GCG GCT CC, and forward ‘LB2F’: AAA TGC CTA CCT GAT CAA CC and reverse ‘LB2R’: CAG ATT ACT TCA ATA TCG CTG T. PCR amplification was performed as described above with an annealing temperature of 54°C. We failed to obtain nuclear PCR products from the age–old herbarium specimens. All PCR products were purified using a kit (Takara, Dalian, China) according to the manufacturer’s protocol. Both forward and reverse strands of the chloroplast and nuclear DNA were sequenced using ABI 3730XL Sequencer. All sequences have been deposited in the DDBJ database with accession numbers LC072680–LC072694 (*trn*L–*trn*F), LC072695–LC072708 (*trn*S–*trn*G), LC072862–LC072866 (products of primers LB1F and LB1R), and LC072867–LC072890 (products of primers LB2F and LB2R).

### Genetic diversity and population differentiation

Haplotype (*h*) and nucleotide (π) diversity at different levels (species, region, and population) were calculated using DnaSP (ver. 5) [23]. A U–statistics test was run in Permut 1.2.1 [24] for the comparison of population differentiation based on ordered (*N*_ST_) and unordered (*G*_ST_) alleles/haplotypes, and the comparison result can be used to investigate whether phylogeographic structure exists at the population level. The genealogical relationships of the cpDNA and nuclear haplotypes were constructed using the median joining (MJ) algorithm in Network 4.6 [25]. In this analysis, indels (gaps) were marked as single mutation events and were coded as substitutions (A or T). Analysis of molecular variance (AMOVA) was also performed to estimate the hierarchical variability within and among populations using Arlequin 3.1 [26].

### Phylogenetic analysis (cpDNA and *rpb*2)

Phylogenetic relationships among the native populations of *L*. *buchtormensis* were inferred using Bayesian inference (BI) and maximum likelihood (ML) analysis, based on cpDNA data and *rpb*2 data, respectively. The best–fit model of nucleotide substitution (cpDNA: TPM1uf + I and *rpb2*: HKY + I) was determined by jModelTest 2.1.5 based on the Akaike information criterion (AIC) [27]. The ML analysis was conducted using PhyML 3.0 [28]. Likelihood bootstrap values (LB) estimated from 1,000 bootstrap replicates were used to assess node support. Bayesian inference was carried out using MrBayes 3.2 [29]. Markov chain Monte Carlo (MCMC) analyses were performed in two independent runs with four chains each for 30,000,000 generations, sampling every 3,000 generations. A 50% majority rule consensus tree with posterior probabilities (PP) was constructed after discarding the first 25% of the sampled trees as burn–in. Because there was no obvious conflict between the topologies recovered from cpDNA and *rpb*2 datasets (see results), we combined these data for phylogenetic analyses (as described above) to improve the resolution of the phylogenetic tree.

### Divergence time estimates and demographic changes

Divergence times were estimated using the cpDNA dataset, with a Bayesian approach as implemented in BEAST 1.8.1 [30]. As neither fossil records nor specific substitution rates of *Libanotis* were available, a mean substitution rate reported for cpDNA sequences was used for calibration. Wolfe et al. [31] estimated a range of 1.0–3.0 × 10^−9^ substitutions per site per year (s/s/y) based on the comprehensive study of chloroplast genes. Considering the uncertainties of the rate values, we assumed a mean of 2 × 10^−9^ s/s/y and a deliberately standard deviation of 6.080 × 10^−10^ s/s/y with a normal distribution prior for the two cpDNA regions (*trn*L–*trn*F and *trn*S–*trn*G) in this study [32,33]. Two independent runs of 1.0 × 10^7^ chains were performed, sampling every 1,000 generations following a 10% burn–in in each chain. We used the HKY + I substitution model, a strict clock, a constant population size coalescent tree prior, and a UPGMA starting tree. Tracer 1.4 was used to confirm the sampling adequacy and convergence of the MCMC output parameters [34]. Both log and tree files from the two independent runs were combined in LogCombiner 1.8.1 (within BEAST). The isolation with migration (IM) coalescent model in IMa [35] was used to estimate the divergence time between the northwestern and southeastern populations. A four years generation time (L. R. Xu, pers. obsv.), the HKY model of sequence evolution, and aforementioned chloroplast substitution rate were employed in the analysis. To verify convergence of samples, three independent runs were performed with different random number seeds.

We conducted mismatch distribution analyses based on the demographic expansion model in Arlequin to detect independent demographic expansion events in the regional populations of *L*. *buchtormensis*. The sum of squared deviations (*SSD*) and Harpending’s raggedness index (*H*_*Rag*_) were chosen to test the validity of the expansion model and quantify the smoothness of mismatch distributions [26,36]. For the expanding group identified, the formula *T* = *τ*/2*u* was used to estimate the expansion time, where *T* is the expansion time in number of generations, *τ* is the expansion parameter detected by the mismatch distribution, and *u* is the neutral mutation rate of the entire cpDNA sequences per generation [37]. The value of *u* was calculated as *u* = μ*kg*, where μ is the substitution rate per nucleotide site per year (assuming 2 × 10^−9^ s/s/y in the study, as mentioned above), *k* is the average sequence length of the analysed cpDNA region, and *g* is the generation time in years (approximated as 4 years).

### Present and past distribution modelling

To infer the distributional dynamics of *L*. *buchtormensis* since the Last Glacial Maximum (LGM: *c*. 21,000 yr before present; BP), a species distribution model was run in MAXENT 3.3.1 using the maximum entropy method [38]. In addition to the distribution records in this study, the records sourced from the Chinese Virtual Herbarium (www.cvh.org.cn) and the National Specimen Information Infrastructure of China (www.nsii.org.cn) were also included. To achieve higher accuracy, we excluded misidentified and uncertain herbarium information. Based on a total of 65 records, a current distribution model was developed using seven bioclimatic data layers (bio8, 10, 13, 14, 15, 17, and 19) from the WorldClim dataset at 2.5 arc–min resolution (available at http://www.worldclim.org/download). The seven bioclimatic data with high gain values were selected by a Jackknife Test to avoid highly correlated variables, which may cause potential overfitting [39,40]. Then, this developed model was projected into the paleoclimatic dataset simulated by the Model for Interdisciplinary Research on Climate (MIROC) 3.2 (available at http://www.worldclim.org/download) to infer the sphere of suitable habitat during the LGM. The accuracy of the predicted model was assessed by calculating the area below the receiver operating characteristic curve (AUC) [41].

### Ancestral area reconstructions

Statistical dispersal–vicariance analysis (S–DIVA) was performed to investigate the hypothetical historical biogeographic areas of *L*. *buchtormensis* using RASP 3.1 [42]. Due to the limited samples only from China, and well–supported monophyletic NW and SE lineages (see results), we reconstructed ancestral areas within the NW and SE regions separately. According to its habitats, the geographical distribution of *L*. *buchtormensis* can be categorized into five areas: (A) Altai Mountains (populations 1–8), (B) steppe mountains from Altai Mountains to Western Tienshan Mountains (populations 9 and 10), (C) Western Tienshan Mountains (populations 11–13), (D) Western Sichuan Plateau (populations 14 and 15), and (E) Qinling Mountains (populations 16–21). The MCMC output and condensed tree exported from BEAST were loaded as ‘trees file’ in this analysis.

## Results

### Plastid DNA sequences

**Fig 2:**
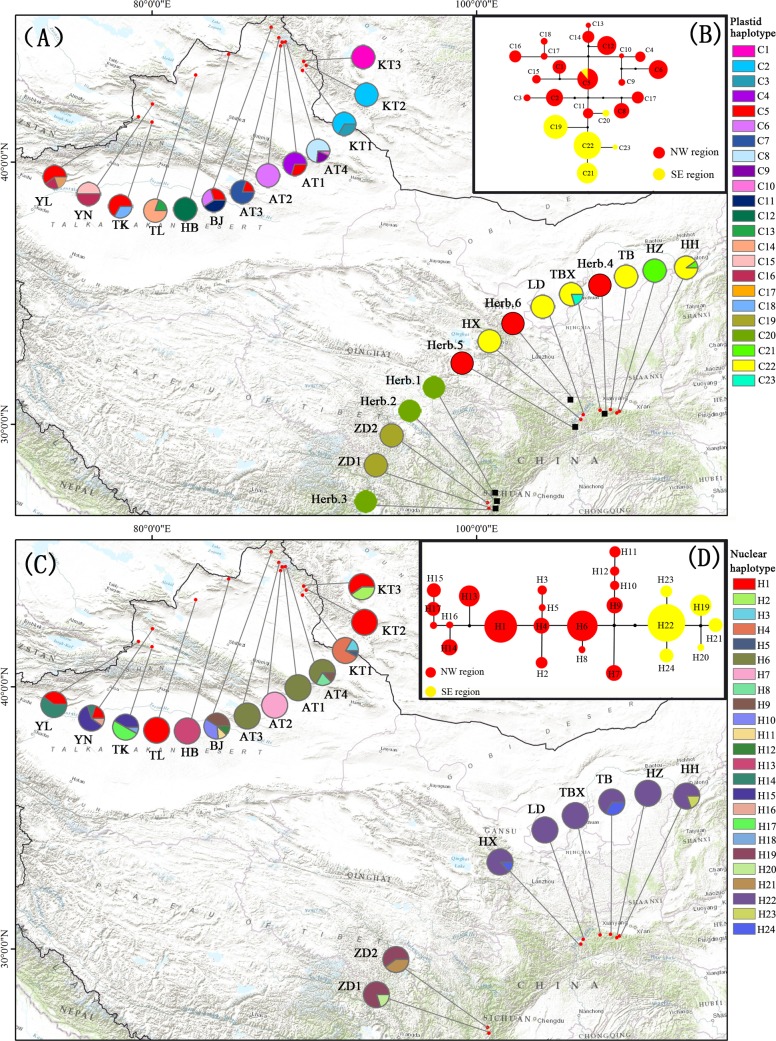
Geographic distribution and relationships of the chloroplast and nuclear haplotypes detected in *Libanotis buchtormensis*. (A) Distribution of 23 plastid haplotypes from 21 natural populations (with red dots) and six herbarium specimens (with black boxes). (B) Median–joining network of the 23 plastid haplotypes. Solid black circles represent the hypothetical missing haplotypes. The size of circles (in red and yellow color) corresponds to the frequency of each haplotype. (C) Distribution of 24 nuclear haplotypes from 21 natural populations (with red dots). (D) Median–joining network of the 24 nuclear haplotypes.

The *trn*L–*trn*F and *trn*S–*trn*G regions were both successfully amplified and sequenced in the 262 individuals from 21 natural populations and six herbarium specimens of *Libanotis buchtormensis*. The total alignment of the combined plastid regions was 1333 bp long. In total, 23 plastid haplotypes were identified based on 19 polymorphic sites (Table 1, S1 Table, Fig 2A). Most of the plastid haplotypes were region specific, and only C5 was shared across the desert zone, occurring in populations from both the NW (populations AT1, AT3, BJ, YL, and TK) and the SE regions (Herb. 4–6) (Table 1, Fig 2A). The NW region had the highest haplotype diversity, with a total of 18 plastid haplotypes recovered from its populations (Table 1). Only four plastid haplotypes were detected in the SE region, and populations ZD1 and ZD2 from the Sichuan Plateau were fixed for a single plastid haplotype (C19). The plastid haplotype network (Fig 2B) showed a close relationship between cpDNA haplotypes, and plastid haplotypes of the SE populations somewhat overlapped with those of the NW populations. Plastid haplotype C5 occupied a central position in the network. Four plastid haplotypes (C19, and C21–23), derived from eight natural populations in the SE region, were only one mutational step from plastid haplotype C11 of the BJ population within the NW region. Except for the shared plastid haplotype C5, another plastid haplotype (C20) found in herbarium species was derived from plastid haplotype C11.

### Nuclear DNA sequences

For nuclear *rpb*2, the amplification and sequencing in six hebarium specimens were failed. From 21 natural populations sampled, we obtained 256 sequences, and the total aligned length was 872 bp. 24 nuclear haplotypes were recovered with 21 substitutions and one indel (Table 1, S2 Table, Fig 2C). No haplotypes were shared between NW and SE regions. A total of 18 nuclear haplotypes were presented in 13 populations within NW region. Populations BJ and YN harbored the greatest number of haplotypes, followed by KT1, AT4 and TK. Nuclear haplotype H1 was detected in five populations (KT2, KT3, TL, YN and YL), representing three geographical areas (A, B and C). Six haplotypes were observed in populations within SE region. Haplotype H19 spanned over all the six populations in QL region, and H22 was observed in the two populations in SC region. The nuclear haplotype network (Fig 2D) grouped the haplotypes from *L*. *buchtormensis* populations into two distinct clades. The six haplotypes detected in SE region resided at the base of the phylogeny.

### Genetic diversity and population structure

High levels of haplotype diversity (*h*_cp_ = 0.909 ± 0.009 and *h*_nr_ = 0.886 ± 0.012) and nucleotide diversity (π_cp_ = 0.0033 ± 0.00055 and π_nr_ = 0.0037 ± 0.0009) were revealed at the species level in *L*. *buchtormensis* (Table 1). At the regional level, nucleotide diversity within the NW populations (*h*_cp_ = 0.921 ± 0.009 and *h*_nr_ = 0.883 ± 0.013) was higher than that within the SE populations (*h*_cp_ = 0.606 ± 0.033 and *h*_nr_ = 0.561 ± 0.048). Population level analysis of sequence data revealed that the BJ, YN, YL, and TK populations in the NW region had the highest genetic diversity (Table 1). With a *G*_ST_–value of 0.671, the cpDNA sequences indicated a high level of differentiation among populations [43]. The level of *N*_ST_ (0.820) was significantly greater than *G*_ST_ (0.671), suggesting a distinct phylogeographical signal in *L*. *buchtormensis*.

At the species level, non–hierarchical AMOVA revealed a strong population genetic structure for the cpDNA sequence variation (*F*_ST_ = 0.848, *P* < 0.0001; Table 2). Hierarchical AMOVA based on cpDNA data revealed that only 11.3% of the total variance was distributed within populations (*F*_ST_ = 0.887, *P* < 0.0001). A large proportion of variation was attributable to differentiation among geographical regions (51.6%, *F*_CT_ = 0.516, P < 0.0001) and between populations within geographical regions (37.1%, *F*_SC_ = 0.766, *P* < 0.0001; Table 2). Nuclear *rpb*2 data revealed a similar pattern of genetic differentiation (Table 2).

**Table 2:** Non–hierarchical and hierarchical analysis using AMOVA based on cpDNA and *rpb*2 sequences of 256 individuals.

Source of variation	d.f.	cpDNA			*rpb*2		
		%Total variance (%)	*F*–statistic	*P*–value	%Total variance (%)	*F*–statistic	*P*–value
Non-hierarchical							
Among Pops	20	84.84	*F*_ST_ = 0.848	< 0.0001	79.84	*F*_ST_ = 0.798	< 0.0001
Within Pops	235	15.16			20.16		
Hierarchical (NW region vs. SE region)							
Among Regions	1	51.58	*F*_CT_ = 0.516	< 0.0001	42.21	*F*_CT_ = 0.422	< 0.0001
Among Pops	19	37.09	*F*_SC_ = 0.766	< 0.0001	41.8	*F*_SC_ = 0.723	< 0.0001
Within Pops	235	11.33	*F*_ST_ = 0.887	< 0.0001	15.99	*F*_ST_ = 0.840	< 0.0001

### Phylogenetic relationships

**Fig 3:**
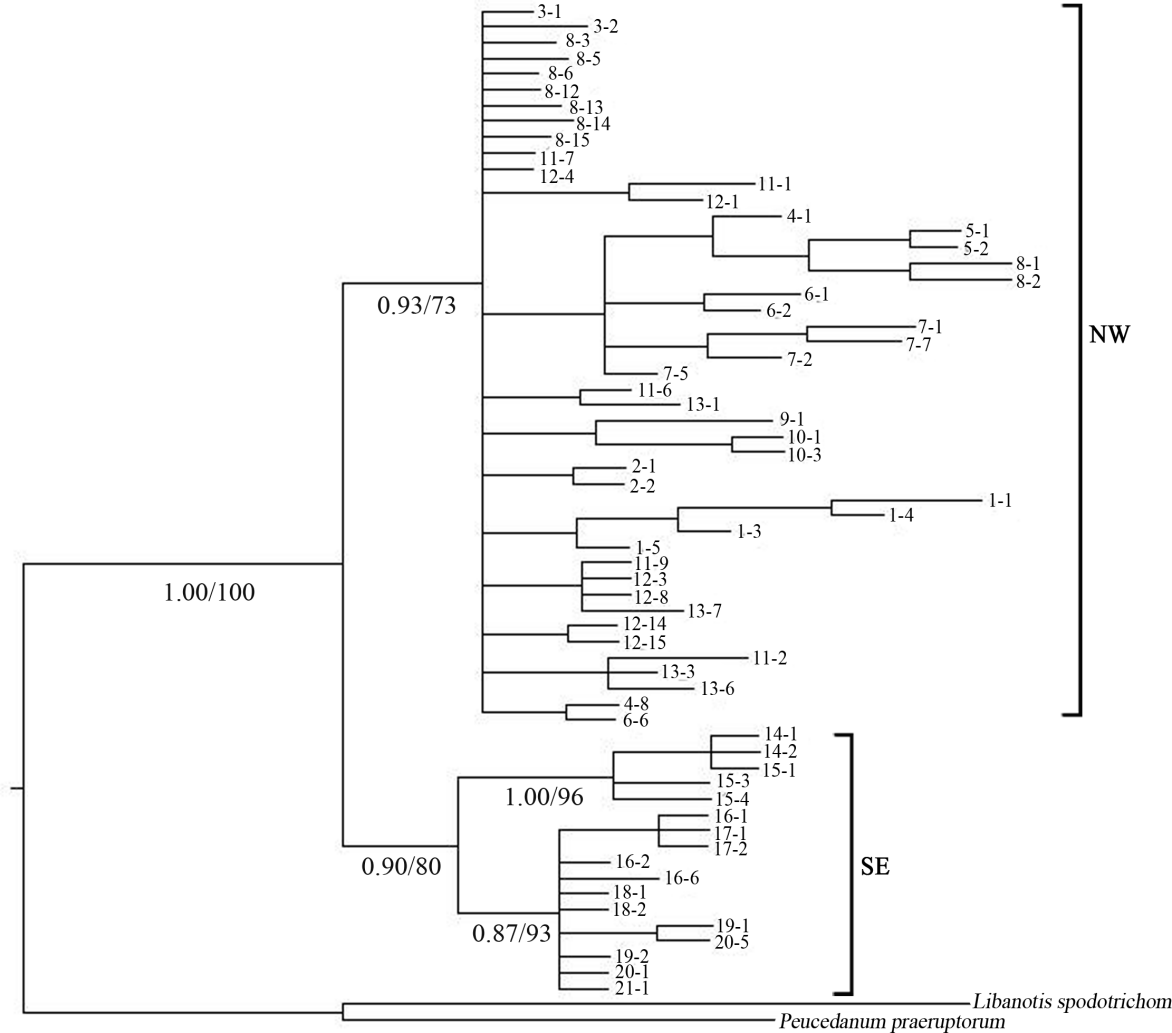
Bayesian 50% consensus tree based on concatenated chloroplast and nuclear *rpb*2 sequences of 63 representative samplings from the 21 *Libanotis buchtormensis*. Numbers below the branches were posterior probabilities (PP) and bootstrap values (LB) for main clades.

The ML and BI trees resulted in similar topologies. BI trees with PP and LB values, based on 63 representative samples from the 21 *L*. *buchtormensis* populations, were presented in Fig 3, S1 and S2 Figs. NW region samples clustered into a group in the plastid tree (S1 Fig), whereas SE region samples formed a clade in the nrDNA tree (S2 Fig), both with low support values. Combined plastid and nuclear data improved the resolution of the phylogenetic tree. The reciprocal monophyly of the NW region samples and the SE region samples were moderately supported in the combined dataset (Fig 3). Clade 1, covering all samples from Xinjiang, represented the northwestern lineage along the desert zone (NW region). Clade 2 included the remaining populations occupying the southeastern lineage of the deserts in the Qinling Mountains and Western Sichuan Plateau (SE region).

### Divergence time estimates and the possible biogeographic analyses

**Fig 4:**
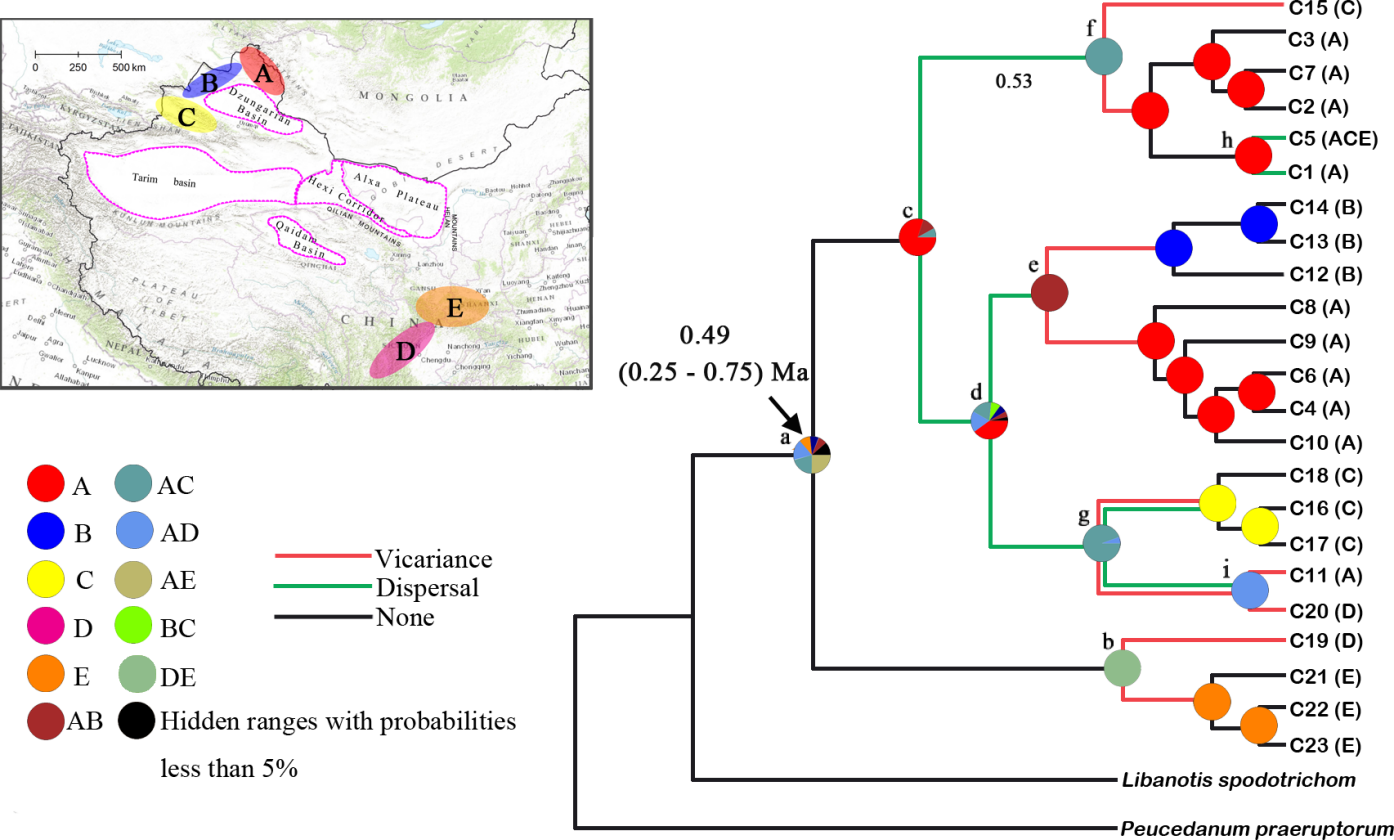
BEAST–derived phylogenetic relationships of 23 plastid haplotypes (C1–C23), divergence dating and ancestral area reconstruction of *Libanotis buchtormensis*. Pie Charts with colors indicate the proportion of the ancestral ranges based on RASP analysis. The red lines and blue lines indicate the vicariance and long–distance dispersal events, respectively. The insert map shows the five major distribution areas (A–E) of *L*. *buchtormensis* in China.

The BEAST analysis (Fig 4) showed that the basal split between clades containing four haplotypes from SE region (C19, C21-23) and the remaining haplotypes occurred at 0.49 (95% HPD: 0.25–0.75) Ma BP, durig the middle Pleistocene (Fig 4). The IMa analysis estimated that the divergence time between the northwestern and southeastern populations was dated to approximately 0.51 (90% HPD: 0.23–0.83) Ma BP, which also falled in the middle Pleistocene.

Regarding the NW lineage, the S–DIVA analysis identified that the most likely ancestral range was the Altai Mountain (A). Dispersal events from the Altai Mountains (A) to the Tienshan Mountains (C) and the steppe mountains from the Altai Mountains to the Western Tienshan Mountains (B) were detected (nodes c, f, e, and g; Fig 4). Meanwhile, several vicariance signals, detected on nodes e and f, indicated that the vicariance events occurred between different mountain ranges. For the SE lineage, a distinctive vicariance event was revealed between the Western Sichuan Plateau (D) and the Qinling Mountains (E) (node b; Fig 4).

### Population demographic history based on cpDNA sequence variation

**Fig 5:**
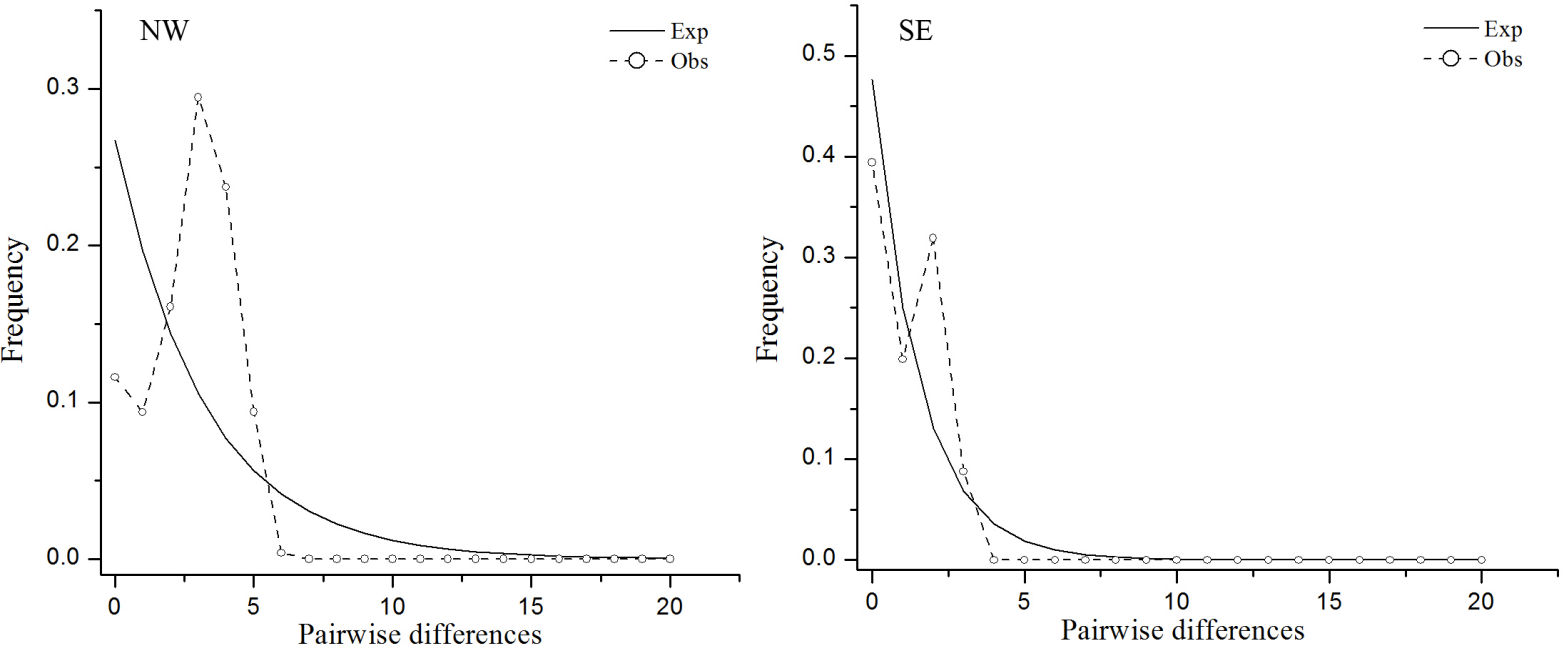
Mismatch distribution of two regional populations (the NW and SE populations) based on chloroplast DNA sequences. The dashed line indicates observed values while solid line reflects expected values.

The unimodal distribution pattern obtained from the mismatch analysis suggested that both the NW and SE populations underwent recent expansions (Fig 5). Non–significant *SSD* statistics and *H*_Rag_ values also favored population expansion events (P > 0.05; Table 3). Based on the corresponding τ value, and assuming cpDNA mutation rates of 2 × 10^−9^ s/s/y, the expansion of the NW and SE populations advanced 107 (95% CI = 55.3–516.3) and 20.4 (95% CI = 0–460.5) ka BP, respectively.

**Table 3:** Results of the mismatch distribution analysis at regional level.

Lineage	τ	expansion time (ka BP)	95% CI	*SSD*	*P*	*H*_*Rag*_	*P*
NW	2.21229	107	55.3–516.3	0.06979	0.09369	0.3906	0.28392
SE	0.427	20.4	0–460.5	0.04188	0.06813	0.07623	0.08762

### Present and past ecological niche models

**Fig 6:**
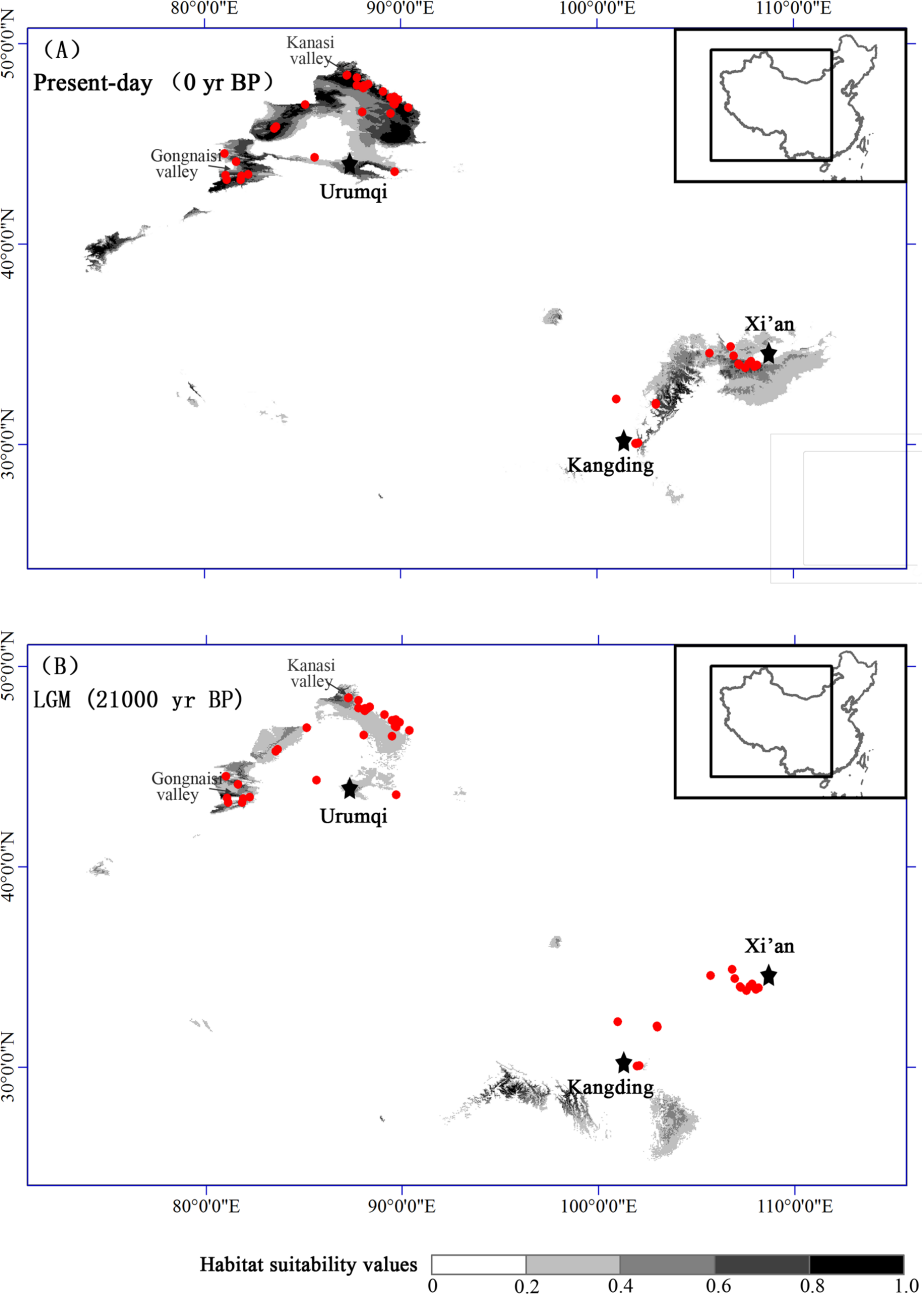
Potential distributions as probability of occurrence for *Libanotis buchtormensis* in China during (a) present day and (b) at the Last Glacial Maximum periods (LGM) based on MIROC model using Maxent.

The high AUC value (0.977) for the present potential distribution of *L*. *buchtormensis* showed a good predictive model performance. The present–day predicted distribution of the species was similar to the actual distribution. Under the simulated LGM climate scenario, we found that most habitats suitable for *L*. *buchtormensis* were reduced relative to present day conditions (Fig 6). In the NW region, the projected current range shrank significantly, and only population locations in the northwestern part of the Altai Mountains and the western part of the Tienshan Mountains remained more or less stable. At the level of resolution used and under the assumptions of the model there was no suitable area inferred for SE populations during the LGM, and SE populations seemed to undergo a southwestward range shift (Fig 6).

## Discussion

### High genetic differentiation due to vicariance

Previous study based on 10 ISSR markers revealed comparatively high genetic diversity of *Libanotis buchtormensis* at the species level. The genetic differentiation among populations contributed to the total genetic divergence [14]. Significant phylogeographical structure and a high level of genetic differentiation were further tested in this study. Phylogenetic relationships showed that *L*. *buchtormensis* of China comprised two geographically distinct lineages, the northwestern lineage distributed in the Xinjiang region (NW region) and the southeastern lineage occupying both the Qinling Mountains and the Sichuan region (SE region). In the nuclear haplotype network, this well–defined genetic structure was recovered as well (Fig 2D). AMOVA analysis also evidenced high differentiation among groups, contributing greatly to the total genetic diversity. The results illustrated a significant phylogeographic break within *L*. *buchtormensis* associated with the desert zone. The pattern of differentiation revealed in this study corroborated our previous result based on ISSR analysis [14].

Two distinct genetic lineages were identified in the geographically disjunct populations of *L*. *buchtormensis*, suggesting that gene flow across the desert zone has been interrupted over a long period of time. The low level of gene flow (*Nm* = 0.22) estimated among the *L*. *buchtormensis* populations evidenced this [14]. Considering the entomophilous pollination and seed dispersal characteristics, *L*. *buchtormensis* is generally incapable of long–distance dispersal, except maybe on rare occasions. Thus, the desert zone in Northwest China has a pivotal effect on the genetic differentiation of *L*. *buchtormensis*, acting as a geographical barrier. We likely proposed a vicariance scenario to explain the current disjunct distribution of *L*. *buchtormensis*. The genetic structure, along with morphological variation such as leaf texture and fruit shape in populations of the two regions (NW and SE), may imply subspecies formation as a result of geographical isolation. Further work is needed to test this.

The estimated divergence time for populations of *L*. *buchtormensis* was dated to the middle Pleistocene (Fig 4). Around this period, the Northern Hemisphere experienced the maximum glacial stage, characterised by cold–dry climatic conditions [44,45]. The deserts, located in the interior of Eurasia and controlled by continental dry air masses, expanded considerably during the Pleistocene cooling [3,7]. Many phylogenetic studies of desert species within Northwest China have inferred the barrier effect of the desert on species distribution and differentiation [3,8–10,46]. Drought–tolerant characteristics make it possible for desert species to adapt to the different habitats following desert expansion. These responses have contributed to genetic differentiation and rapid speciation. Due to the climatic and local site conditions, *L*. *buchtormensis* might stands little chance of occupying arid conditions. The expansion of deserts might block the gene flow between the NW and SE populations and contributed to the genetic divergence of *L*. *buchtormensis*. Even after the subsequent glacial retreat, it is likely that populations in the SW region were still not able to traverse the geographic barriers of the deserts, and as a result, the populations were isolated and developed independently in the Qingling Mountains and Sichuan Plateau. The geographical vicariance, the desert zone in Northwest China, likely contributed largely to the disjunct distribution of *L*. *buchtormensis*.

In the present study, the NW lineage had a higher amount of ancestral and unique haplotypes than did the SE lineage (Table 1, Fig 2A and 2C). Most populations in the SE region were fixed for only one plastid haplotype and nuclear haplotype (Table 1, Fig 2A and 2C). Within all plastid haplotypes generated from the 21 natural populations and six herbarium species, plastid haplotype C5 was shared among populations AT1, AT3, BJ, TK, and YL from the NW region and Herb. 4–6 from the SE region (Table 1, Fig 2A). The sharing of plastid haplotype C5 across populations of *L*. *buchtormensis* might indicate the retention of ancestral polymorphisms (i.e., incomplete lineage sorting) [47,48]. Furthermore, the widespread C5 was found to occupy a central position in the plastid haplotype network, which is inferred to be the ancestral haplotype according to coalescent theory [49,50]. The haplotypes from the SE region (C19, and C21–23) occupied ‘tip’ (derived) positions relative to the ancestral plastid haplotype C5 from the NW region. In particular, the two plastid haplotypes (C5 and C20) from the six herbarium specimens of the Qinling Mountains and Western Sichuan Plateau were more closely related to the NW lineage. Although this result should be discussed cautiously due to the few individuals of herbarium specimens investigated, it provided us, at the least to some degree, an additional suggestion about the historical connection between the NW and SE populations of *L*. *buchtormensis*. These findings indicated that the SE populations might have originated from ones in the NW region by occasional dispersal events. However, the genetic imprint of the founding event may not be detectable after a few generations, especially when anthropogenic destruction exists [51]. According to field observations and specimen information, the populations are widely distributed in Xinjiang Province (NW region), while they are difficult to discover in the SE region, especially in Sichuan province. Additionally, the low genetic diversity and fixed haplotypes of SE populations were also detected in the study. The distribution pattern suggested that the SE region likely to represent peripheral populations of the geographical range in China.

### Glacial refugia and demographic history of *L*. *buchtormensis* in China

Following the initial genetic divergence of the NW and SE lineages, the current distribution of *L*. *buchtormensis* would have been further influenced by the subsequent glacial retreat to refugia and interglacial recolonization. The NW region was identified as the main centre of genetic variation and haplotype endemism. Refugia are expected to possess high levels of genetic diversity and haplotype uniqueness [52]. Compared with the current species range, the range of *L*. *buchtormensis* contracted dramatically during LGM, and it was restricted to the western Altai Mountains and western Tienshan Mountains (Fig 6). Influenced by westerly winds, a high degree of precipitation is received in the mid–elevation valleys. The humid areas located in the western parts of the Altai and Tienshan Mountains (i.e., Kanasi valley and Gongnaisi valley) likely served as refugia for *L*. *buchtormensis* during the glacial intervals, where the four representative population sites (BJ, YN, YL, and TK) harbored higher levels of genetic diversity and the greatest number of unique haplotypes. The refugia identified have also been supported for other plant species in the area [3,53,54]. The glacial retreat response was different from that of the desert species which exhibited population growth following desert expansion during the arid glacial periods [3,10]. Multiple local refugia in Xinjiang Province provided shelters for species survival during arid–glacial periods.

The unimodal mismatch distribution (Fig 5) revealed a significant recent range expansion within the NW region at about 107 ka BP, coinciding with the previous interglacial period following the penultimate glacial cycle in Northwest China (*c*. 136–76 ka BP) [45]. We should keep in mind that the estimate should be treated with caution considering the wide confidence interval. We inferred that the current widespread distribution of plastid haplotype C5 resulted from the expansion of these refugia with the warming process during interglacial periods. The S–DIVA analysis (Fig 4) identified the Altai Mountain as very likely ancestral range, from which to colonize the Tienshan Monutains and steppe mountains along the route with two independent dispersal events. The vicariance events between these mountain ranges may further have contributed to the extant discrete distributions of *L*. *buchtormensis* in the NW region [14].

Regarding the SE lineage, populations in the region expanded their range at 20.4 ka BP, approximately coinciding with the second Taibai glacial period in East Asia (*c*. 19–11 ka BP) [45]. The timing was consistent with two possible range expansion events in north vs. south of the Qinling Mountains, even though this was a glacial period [55]. Although a southward retreat to the Hengduan Mountains was detected in the SE region (Fig 5), we presumed that the expansion range would not extend farther, as no specimen records have been found in the Hengduan Mountains to date. Expected to be the peripheral distribution of *L*. *buchtormensis*, the SE region may have a chance to inherit only a small portion of the genetic diversity present in the ancient parental distribution (NW region). The distribution pattern of *L*. *buchtormensis* in the SE region is island–like to some extent, and characterized by low genetic diversity. Studies on island populations have shown that long–term isolated and small habitat sized populations would be threatened by genetic drift and inbreeding [47,56]. A significant amount of inbreeding and the contemporary anthropogenic over–exploration have resulted in the genetic diversity loss and fragmentation of *L*. *buchtormensis* in the Qinling Mountains and Sichuan Plateau [14]. Thus, in this study, we reiterate that it is urgent to take measures to protect the species in the SE region from being further endangered.

## Supporting Information

S1 Fig Bayesian consensus tree of *Libanotis buchtormensis* based on chloroplast DNA regions (*trn*L–*trn*F and *trn*S–*trn*G). Numbers below the branches were BI posterior probabilities (PP) and ML bootstrap values (LB), respectively. The dash lines indicated that the branches were not supported in the phylogenetic analyses.
(TIF)

S2 Fig Maximum likelihood (ML) phylogram of *Libanotis buchtormensis* based on nuclear gene (*rpb*2). Numbers below the branches were BI posterior probabilities (PP) and ML bootstrap values (LB), respectively. The dash lines indicated that the branches were not supported in the phylogenetic analyses.
(TIF)

S1 Table Variable sites of 23 plastid haplotypes (C1–C23) in two chloroplast DNA regions (trnL–trnF and trnS–trnG) generared from Libanotis buchtormensis. All sequences are compared to the reference plastid haplotype C1.
(DOC)

S2 Table Variable sites of 24 nuclear haplotypes (H1–H24) in *rpb*2 gene generared from *Libanotis buchtormensis*. All sequences are compared to the reference haplotype H1.
(DOC)
