# Supplementary figures and images for: Phylogeography of Libanotis buchtormensis (Umbelliferae) in Disjunct Populations along the Deserts in Northwest China
Source: PLoS One. 2016 Jul 21;11(7):e0159790. doi: 10.1371/journal.pone.0159790 (PMC4956107; doi:10.1371/journal.pone.0159790)

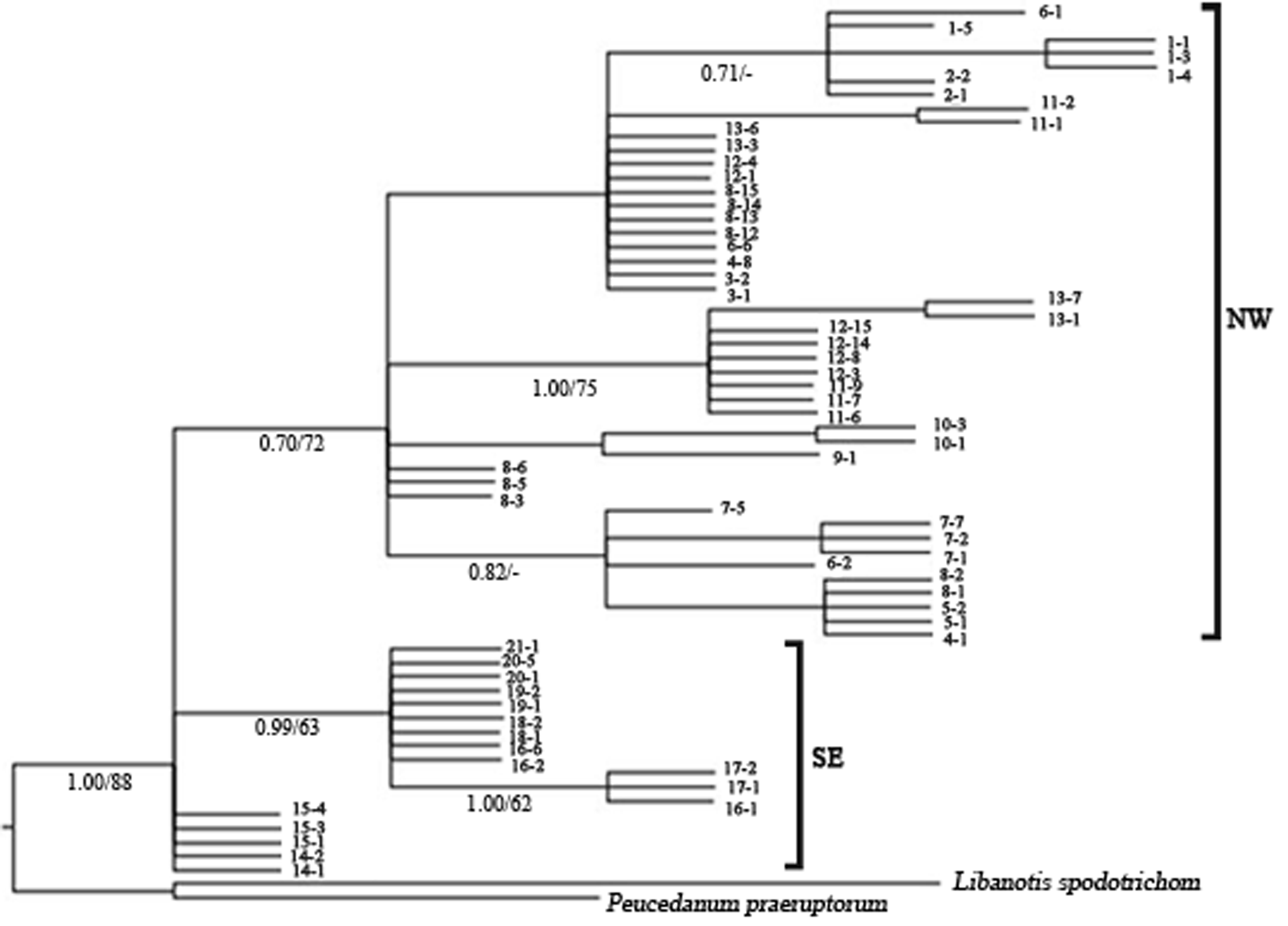

Supplement: S1 Fig — Numbers below the branches were BI posterior probabilities (PP) and ML bootstrap values (LB), respectively. The dash lines indicated that the branches were not supported in the phylogenetic analyses. (TIF) [file pone.0159790.s001.tif]

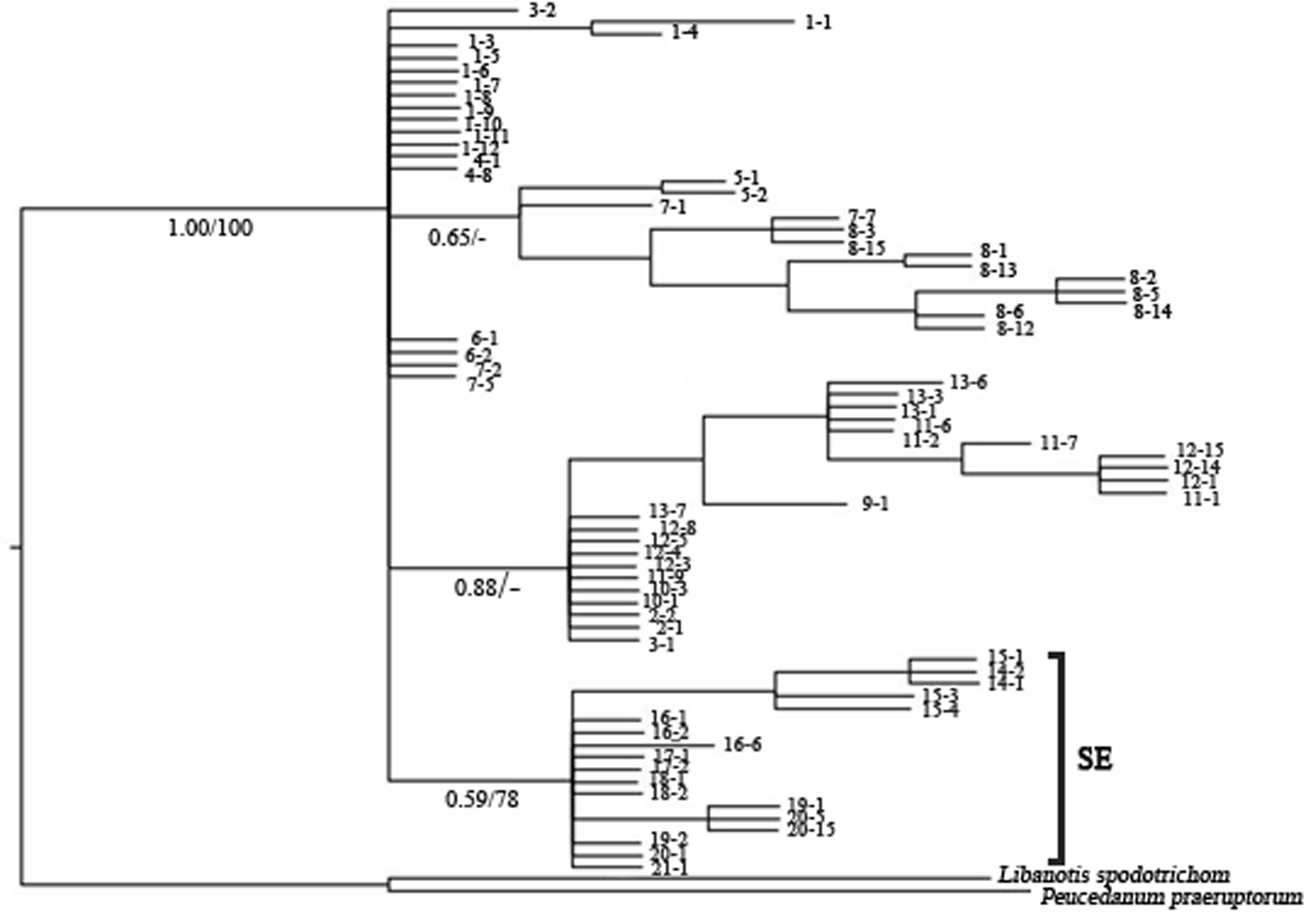

Supplement: S2 Fig — Numbers below the branches were BI posterior probabilities (PP) and ML bootstrap values (LB), respectively. The dash lines indicated that the branches were not supported in the phylogenetic analyses. (TIF) [file pone.0159790.s002.tif]
