# Supplementary material for: Phylogeography of Libanotis buchtormensis (Umbelliferae) in Disjunct Populations along the Deserts in Northwest China
Source: PLoS One. 2016 Jul 21;11(7):e0159790. doi: 10.1371/journal.pone.0159790 (PMC4956107; doi:10.1371/journal.pone.0159790)
Supplement: S1 Table — All sequences are compared to the reference plastid haplotype C1. (DOC) [file pone.0159790.s003.doc]

**S1 Table.** Variable sites of 23 plastid haplotypes (C1-C23) in two chloroplast DNA regions (*trn*L-*trn*F and *trn*S-*trn*G) generared from *Libanotis buchtormensis*. All sequences are compared to the reference plastid haplotype C1.

|  | *trn*L-F | | | | | | | | | | *trn*S-G | | | | | | | | |
| --- | --- | --- | --- | --- | --- | --- | --- | --- | --- | --- | --- | --- | --- | --- | --- | --- | --- | --- | --- |
| Chlorotype | 86 | 1  14 | 1  23 | 2  72 | 2  90 | 3  46 | 5  68 | 5  89 | 7  26 | 8  91 | 9  33 | 1  00  4 | 1  0  24 | 1  0  42 | 1  0  43 | 1  0  64 | 1  0  65 | 1  2  19 | 1  2  94 |
| C1 | * | G | A | G | - | G | G | # | G | & | G | G | T | A | - | T | G | T | G |
| C2 | - | . | . | . | - | . | . | . | A | - | . | . | . | . | - | . | . | . | . |
| C3 | - | . | . | . | - | . | T | . | A | - | . | . | . | . | - | . | . | . | . |
| C4 | . | . | . | . | - | . | . | . | . | - | . | C | G | T | - | . | . | . | . |
| C5 | . | . | . | . | - | . | . | . | . | - | . | . | . | . | - | . | . | . | . |
| C6 | - | . | . | . | - | . | . | . | . | . | . | C | G | T | - | . | . | . | . |
| C7 | - | . | . | . | - | . | . | . | A | . | . | . | . | T | - | . | . | . | . |
| C8 | - | . | . | . | - | . | . | . | A | - | . | . | G | T | - | . | . | . | . |
| C9 | - | A | . | . | - | . | . | . | . | - | . | . | G | T | - | . | . | . | . |
| C10 | . | . | . | . | - | . | . | . | . | - | . | . | G | T | - | . | . | . | . |
| C11 | - | . | . | . | - | . | . | . | . | - | . | . | G | . | - | . | . | . | . |
| C12 | . | . | . | . | - | . | . | . | . | - | . | . | G | . | - | . | . | G | A |
| C13 | - | . | . | . | - | . | . | . | . | - | . | . | G | . | A | . | T | . | A |
| C14 | . | . | . | . | - | . | . | . | . | - | . | . | G | . | - | . | T | . | A |
| C15 | . | . | G | . | A | . | T | . | . | - | . | . | . | . | - | . | . | . | . |
| C16 | . | . | G | . | - | A | . | . | . | - | . | . | G | . | - | - | . | . | . |
| C17 | . | . | G | . | - | A | . | . | . | - | . | . | G | . | - | . | . | . | . |
| C18 | . | . | G | . | - | A | . | . | . | - | . | . | G | . | - | . | . | G | . |
| C19 | - | . | . | . | - | . | T | - | . | - | . | . | G | . | A | . | . | . | . |
| C20 | - | . | . | . | - | . | . | . | . | . | . | . | G | . | - | . | . | . | . |
| C21 | - | . | . | T | - | . | T | - | . | - | T | . | G | . | - | . | . | . | . |
| C22 | - | . | . | T | - | . | T | - | . | - | . | . | G | . | - | . | . | . | . |
| C23 | - | . | . | T | - | . | T | - | . | - | . | . | G | . | A | . | . | . | . |

* indicates 'TTTTACAAAA';

# indicates 'TTATCTTATCTTTTTTTT';

& indicates 'TAGGTTATCTAGTAAATT';

- indicates gap.
