# Supplementary material for: Phylogeography of Libanotis buchtormensis (Umbelliferae) in Disjunct Populations along the Deserts in Northwest China
Source: PLoS One. 2016 Jul 21;11(7):e0159790. doi: 10.1371/journal.pone.0159790 (PMC4956107; doi:10.1371/journal.pone.0159790)
Supplement: S2 Table — All sequences are compared to the reference haplotype H1. (DOC) [file pone.0159790.s004.doc]

**S2 Table.** Variable sites of 24 nuclear haplotypes (H1-H24) in *rpb*2 gene generared from *Libanotis buchtormensis*. All sequences are compared to the reference haplotype H1.

|  | LBF1-  LBR1 | | LBF2-LBR2 | | | | | | | | | | | | | | | | | | | |
| --- | --- | --- | --- | --- | --- | --- | --- | --- | --- | --- | --- | --- | --- | --- | --- | --- | --- | --- | --- | --- | --- | --- |
| Haplotype | 1  0  7 | 1  0  8 | 1  8  5 | 2  3  0 | 2  6  2 | 3  3  8 | 3  4  5 | 3  4  6 | 3  8  8 | 4  1  0 | 4  2  8 | 4  4  6 | 4  9  4 | 4  9  5 | 4  9  9 | 5  4  7 | 5  6  5 | 5  7  9 | 5  8  0 | 5  9  3 | 6  4  4 | 7  8  1 |
| H1 | T | T | T | T | T | A | C | G | A | A | G | C | A | A | A | A | T | G | C | A | C | G |
| H2 | . | C | . | . | . | . | . | A | . | . | . | . | . | . | . | . | . | . | . | . | . | . |
| H3 | A | C | . | . | . | . | . | A | . | . | . | A | . | . | . | . | . | . | . | . | T | . |
| H4 | . | C | . | . | . | . | . | . | . | . | . | . | . | . | . | . | . | . | . | . | . | . |
| H5 | A | C | . | . | . | . | . | . | . | . | . | A | . | . | . | . | . | . | . | . | . | . |
| H6 | . | C | . | . | . | . | . | . | . | . | . | . | . | . | . | . | . | A | . | . | . | . |
| H7 | . | C | . | . | C | . | . | . | . | C | . | . | . | . | . | . | . | A | . | . | . | . |
| H8 | . | C | . | . | C | . | . | A | . | . | . | . | . | . | . | . | . | A | . | . | . | . |
| H9 | . | C | . | . | C | . | . | . | . | . | . | . | . | G | . | . | . | A | . | . | . | . |
| H10 | . | C | . | . | C | . | . | . | . | . | . | . | . | G | . | . | . | A | . | . | . | T |
| H11 | . | . | . | . | C | . | . | . | . | . | . | . | . | . | . | . | . | A | . | . | . | T |
| H12 | . | C | . | . | C | . | . | . | . | . | . | . | . | . | . | . | . | A | . | . | . | T |
| H13 | . | . | . | . | C | . | . | . | . | . | . | . | . | . | . | . | . | . | . | . | T | . |
| H14 | . | . | . | . | C | . | . | . | . | . | A | . | . | . | C | . | . | . | . | . | . | . |
| H15 | . | . | . | . | C | . | . | . | . | . | . | . | . | . | . | - | . | . | . | T | . | . |
| H16 | . | . | . | . | C | . | . | . | . | . | . | . | . | . | C | . | . | . | . | . | . | . |
| H17 | . | . | . | . | C | . | . | . | . | . | . | . | . | . | . | . | . | . | . | T | . | . |
| H18 | . | . | . | . | C | . | . | . | . | . | . | . | . | . | C | . | . | . | . | T | . | . |
| H19 | . | C | C | . | C | . | T | . | G | . | . | . | T | . | . | . | . | . | . | . | . | . |
| H20 | . | C | C | . | C | G | T | . | . | . | . | . | T | . | . | . | . | . | . | . | . | . |
| H21 | . | C | C | . | C | . | T | . | . | . | . | . | T | . | . | . | . | . | T | . | . | . |
| H22 | . | C | . | . | C | . | . | . | . | . | . | . | T | . | . | . | . | . | . | . | . | . |
| H23 | . | C | . | . | C | . | . | . | . | . | . | . | T | . | . | . | C | . | . | . | . | . |
| H24 | . | C | . | C | C | . | . | . | . | . | . | . | T | . | . | . | . | . | . | . | . | . |

- indicates gap.
